# A method for continuous study of soaring and windhovering birds

**DOI:** 10.1038/s41598-022-10359-w

**Published:** 2022-04-29

**Authors:** Matthew Penn, George Yi, Simon Watkins, Mario Martinez Groves-Raines, Shane P. Windsor, Abdulghani Mohamed

**Affiliations:** 1 RMIT University, Melbourne, Australia; 2 University of Bristol, Bristol, UK

**Keywords:** Aerospace engineering, Animal behaviour

## Abstract

Avian flight continues to inspire aircraft designers. Reducing the scale of autonomous aircraft to that of birds and large insects has resulted in new control challenges when attempting to hold steady flight in turbulent atmospheric wind. Some birds, however, are capable of remarkably stable hovering flight in the same conditions. This work describes the development of a wind tunnel configuration that facilitates the study of flapless windhovering (hanging) and soaring bird flight in wind conditions replicating those in nature. Updrafts were generated by flow over replica “hills” and turbulence was introduced through upstream grids, which had already been developed to replicate atmospheric turbulence in prior studies. Successful flight tests with windhovering nankeen kestrels (*Falco cenchroides*) were conducted, verifying that the facility can support soaring and wind hovering bird flight. The wind tunnel allows the flow characteristics to be carefully controlled and measured, providing great advantages over outdoor flight tests. Also, existing wind tunnels may be readily configured using this method, providing a simpler alternative to the development of dedicated bird flight wind tunnels such as tilting wind tunnels, and the large test section allows for the replication of orographic soaring. This methodology holds promise for future testing investigating the flight behaviour and control responses employed by soaring and windhovering birds.

## Introduction

Unmanned air vehicles (UAVs) are challenged to hold steady flight in windy conditions, whereas birds are known to maintain far steadier flight under a range of flight conditions. Several species of birds, including ospreys, kestrels, and pied kingfishers, can hover at low altitudes when flying into the wind, essentially keeping their heads stationary relative to the Earth’s surface as they watch for small prey moving below^1–3^. This flight behaviour is termed *windhovering*. Windhovering without wing flapping, termed *hanging*, can occur when birds are in the presence of suitable orographic updrafts (i.e. hill soaring)^4^. This entails coping with the significant turbulence inherent in the atmospheric boundary layer (ABL)^5^. Kestrels have been observed to hang with their heads deviating from a fixed position by less than 6 mm despite the turbulent nature of the wind^1^. The control mechanisms and sensing strategies used to maintain this stability are not understood. Studies investigating the control strategies employed by windhovering birds would likely discover novel gust-mitigation techniques that inspire the development of ultra-stable UAVs, a concept that has been the subject of recent research^6–15^.

Whilst the aerodynamics of windhovering flight has been studied outdoors^4^, there have been no studies of windhovering flight in controlled smooth and turbulent wind flows. It is considered that this is due to the lack of a suitable facility which would permit windhovering studies. Tilting wind tunnels have been used to study the gliding flight of a range of birds^16–21^, however, these studies focus on aerodynamic performance in smooth flow rather than investigating control responses to gusts and turbulence. Such responses in flapping flight have been the focus of several studies involving hummingbird flight through gusts or turbulence^22–24^, however, wing flapping confounds the extraction of the disturbance mitigation control kinematics. Cheney et al.^25^ studied gliding flight of a barn owl through discrete vertical gusts in still air, but this is a different situation from soaring and hanging flight in moving air with continuous turbulence, which is more representative of fixed-wing flight through atmospheric turbulence. This work describes modifications to a wind tunnel environment to enable soaring and windhovering flight behaviours of birds to be studied in a range of replicated outdoor conditions. Initial results of flight trials with nankeen kestrels (*Falco cenchroides*) are also described to illustrate the utility of this approach. There has been much recent interest in the research of bird flight in controlled conditions. This methodology provides an opportunity for many new discoveries into the behaviour, aerodynamics and biomechanics of bird flight.

### The windhovering flow environment

To replicate the flow conditions in which birds naturally windhover it is necessary to understand several key properties of the flow, including windspeeds, updraft angles and turbulence intensities. The typical flow updraft angle preferred by hanging kestrels can be obtained from the work of Videler and Groenewold^4^ where a glide angle between 6 and 7° in flight speeds of 7 to 12 m/s was reported for kestrels windhovering over a dike in the Netherlands. These estimates were obtained by correlating windhover locations to wind profiles constructed from a set of 225 measurements of mean wind speeds and angles over the dike at 13 different heights. Ospreys have been observed to windhover with gliding flight in windspeeds between 7 and 15 m/s, although the glide angles are unknown. Table 1 reports data from wind tunnel tests conducted with a range of gliding birds and bird-sized UAVs (UAVs were mounted on a sting). The table reports the velocity range in which tests were conducted, as well as the maximum lift-to-drag ratio (L/D). The minimum glide angle was calculated from L/D values using Eq. (1) below:

**Table 1:** Velocities and glide angles reported from wind tunnel tests with soaring birds and UAVs.

Bird	V_min_ [m/s]	V_max_ [m/s]	V_min sink_ [m/s]	V_θmin_ [m/s]	(L/D)_max_	θ_min_ [°]	Source
Pigeon (*Columbia livia*)	8	22	–	–	6	9.5	^21^
Laggar falcon (*Falco jugger*)	6.6	15.9	–	12.5	10	5.7	^20^
Black vulture (*Coragyps atratus*)	9.9	16.8	11.6	13.9	11.6	4.9	^19^
Harris' hawk (*Parabuteo unicinctus*)	6.1	16.2	8.8	–	10.9	5.2	^18^
Jackdaw (*Corvus monedula*)	6	11	7.4	8.3	12.6	4.5	^17^
Common swift (*Apus apus* L.)	7	11	8.1	9.4	12.5	4.6	^16^
**UAV**							
Astro-mite	6	9.5	–	–	10	5.7	^20^
Dream-flight Alula	4.7	13	5	6.5	12	4.8	^26^

1 $${\theta }_{min}={\mathrm{cot}}^{-1}\left(L/D\right)$$

Flight velocities of birds in Table 1 range between 6 and 16.8 m/s, although the velocities at which minimum sink rate or minimum glide angle occur are between 7.4 and 13.9 m/s. The minimum glide angle for most tests was between 4.5 and 6°, except for the pigeon. The glide angles and velocities reported for the UAVs are very similar to those for the birds. Thus a wind tunnel capable of producing windspeeds between 5 and 17 m/s, and updraft angles between 4.5 and 10° should be capable of facilitating a wide range of studies involving soaring or windhovering birds or UAVs.

**Figure 1:**
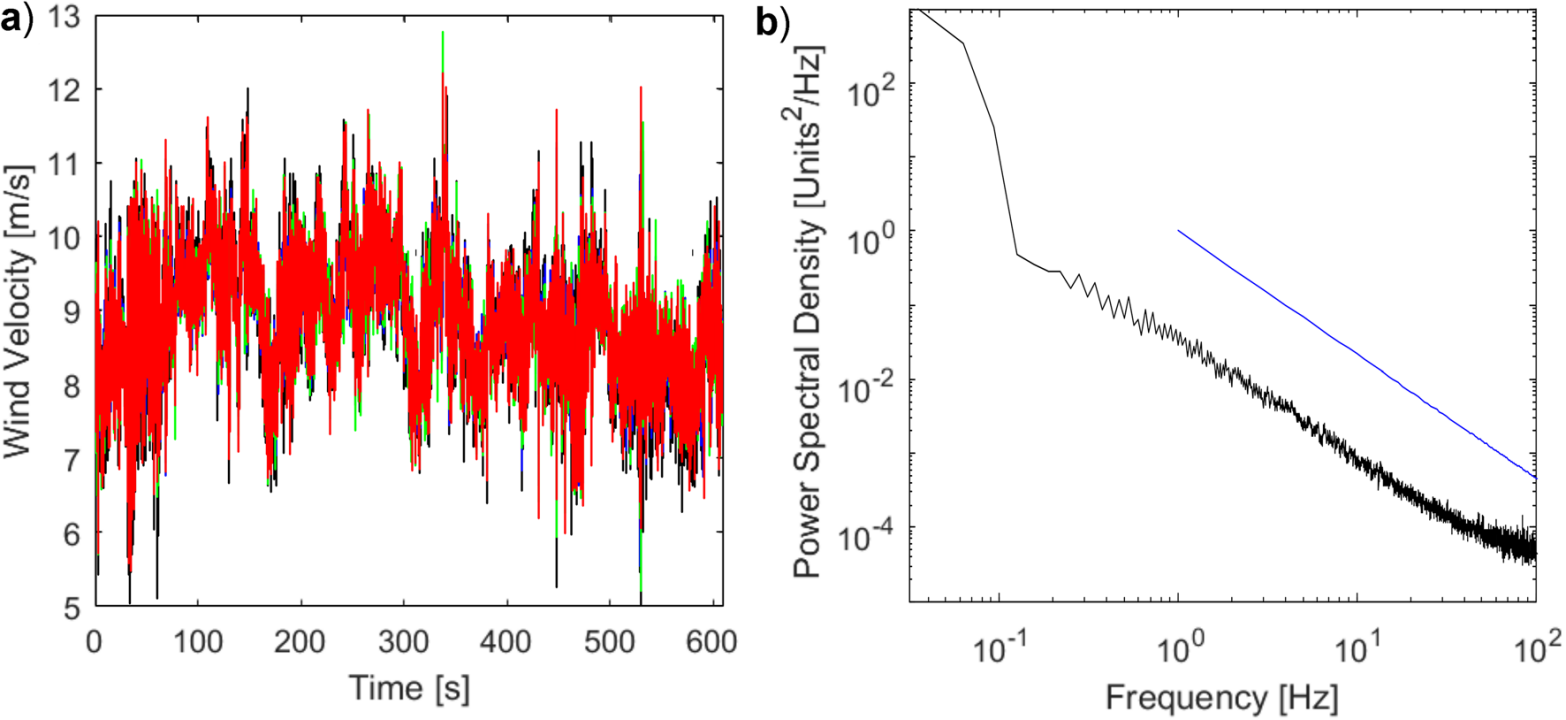
Temporal and spectral characteristics of wind measured at a location on the Gower Peninsula in the UK where birds have been observed to windhover. (**a**) shows a 10 min sample of the fluctuating wind velocity measured by four TFI Cobra Probes. The velocity from each of the four probes is shown in a different colour. (**b**) shows the power spectral density of the flow follows the − 5/3 Kolmogorov spectral decay depicted by the blue line.

To date, there appear to be no dedicated outdoor flow measurements to document the turbulence in which birds windhover, however, several studies have sought to measure the turbulent wind domains for UAVs flying over similar terrains. One such study was conducted by Watkins et al.^27^ who utilised a row of four TFI Cobra Probes mounted on a mast above a stationary and moving car. Cobra probes are commercially available pressure-based velocity measuring instruments that measure u, v and w components of fluctuating airflow velocity. The probes are relatively robust yet still have a flat frequency response to 2 kHz. Details can be found in^28^. This study documented the flow environment experienced by flight through the atmospheric boundary layer. Probes were laterally displaced by 150 mm giving a total span of 450 mm. These measurements of fluctuating velocity components included flow in city, suburban and relatively smooth domains, such as open fields with lightly undulating terrain. They revealed the detailed time-varying flow characteristics relevant to the flight of birds and UAVs. Details can be found in^29^. This work was extended to encompass a range of probe lateral separations, locations and heights up to 10 m above the ground^30^. Later work involved airborne measurements using the Cobra Probes^10,31,32^. Additional hitherto unpublished measurements were taken above sand dunes where birds had been observed to windhover, using the method and instrumentation described by Thompson et al.^33^ and in the thesis by Thompson^30^. The location was on the north side of the Gower Peninsula in the UK and the upstream terrain was smooth (the Atlantic Ocean and a short section of relatively smooth sand). The longitudinal velocities from the four laterally separated Cobra Probes were very similar (within 1 m/s), as seen in the measurement sample provided in Fig. 1a. The spectral characteristics of atmospheric turbulence, when removed from any local wakes that might generate vortex shedding, are known to follow a reduction of energy with increasing frequency (known as the − 5/3rds Kolmogorov spectral decay), as seen in Fig. 1b. This confirms that the flow was well-mixed and free from any local vortex shedding from upstream structures. From eight such data sets, differences in the turbulence characteristics were found to be less than 2% resulting in turbulence intensities that varied from 8 to 10% in mean wind speeds of 6–10 m/s. Other data sets obtained in rougher mountainous terrains (where birds had again been observed to windhover) exhibited turbulence intensities of typically 15%.

Thus a wind tunnel facilitating the study of soaring or windhovering flight in birds or small UAVs should provide a region of flow sufficiently large for birds to windhover in flow speeds ranging from 5 to 17 m/s, time-averaged flow pitch angles in the range 4.5 to 10°, and turbulence intensity levels up to 15%. This would encompass the gliding envelope for the birds and UAVs listed in Table 1.

One possibility for studying continuous gliding flight is to have a dedicated wind tunnel where the test section can be inclined to the glide angle of a bird. This provides an updraft allowing gliding flight to be continuously sustained. Such tilting wind tunnels are described by Pennycuick, Alerstam and Hedenstrom^34^. The requirement to tilt the wind tunnel introduces significant design challenges. Small, open circuit tilting tunnels typically require the bird to fly within a small volume of the flow. Large closed-section tunnels provide the potential for better aerodynamic performance and the study of larger birds, but can be prohibitively expensive to develop. An alternative method of providing an updraft to sustain soaring flight can be achieved through temporary modifications to the test section of an existing conventional tunnel. A ramp or other obstacle can be included in the test section to replicate a “hill” and thus generate an “orographic” updraft in which birds can soar. This is potentially a far cheaper and more rapidly implemented solution, providing researchers with a suitable facility to study soaring bird flight. It should be noted, however, that both methods do not produce identical updrafts. Tilting wind tunnels provide an updraft with near uniform flow properties throughout the test section, while the flow properties of orographic updrafts vary with position relative to the obstacle.

A wind tunnel facility modified in this manner may be used to study the flight behaviour and energetics of soaring and windhovering birds or small UAVs. It may also be used to study the control responses of birds to continuous turbulence or discrete gusts. Discrete gusts have been produced in wind tunnels via a number of different methods. The most common methods involve the use of jets, or rotating/oscillating devices that deflect airflow by generating lift or drag^35^. The addition of one of these devices into the wind tunnel facility would allow a controlled study of the control responses of gliding birds to various flow disturbances. Such studies would shed light on the control kinematics and morphology used by gliding birds, about which very little is currently known. This understanding would also be of great interest to developers of small, fixed-wing UAVs seeking to improve the aircraft’s gust and turbulence mitigating capabilities.

The requirements for a wind tunnel to be modified in this manner are dependent on the application. The use of a large wind tunnel will provide a large volume with flow characteristics suitable to sustain soaring flight. Large test sections also provide the possibility of generating large turbulence length scales. Thus large wind tunnels allow greater freedom of movement for smaller birds, or the possibility of flight tests with large birds in turbulence length scales relevant to their wing size. The specifics of each study will dictate the required flow quality. In general, large, industrial wind tunnels have higher baseline turbulence intensities compared to smaller aerodynamic wind tunnels, however, the turbulence intensities observed in industrial wind tunnels is still far lower than those found in nature. A robust device for measuring flow direction and velocity is also required. This may include multi-holed pressure probes, hot-wire anemometers, or similar.

The RMIT Industrial Wind Tunnel was selected for modifications to simulate the flow conditions in which birds naturally windhover. The size of the tunnel’s test section (3 m × 2 m × 9 m) was considered well-suited to study windhovering bird flight under smooth and turbulent conditions. The relatively long test section gave sufficient distance for the generation of well mixed isotropic turbulence to be generated using planar grids.

## Methods and materials

### RMIT industrial wind tunnel

**Figure 2:**
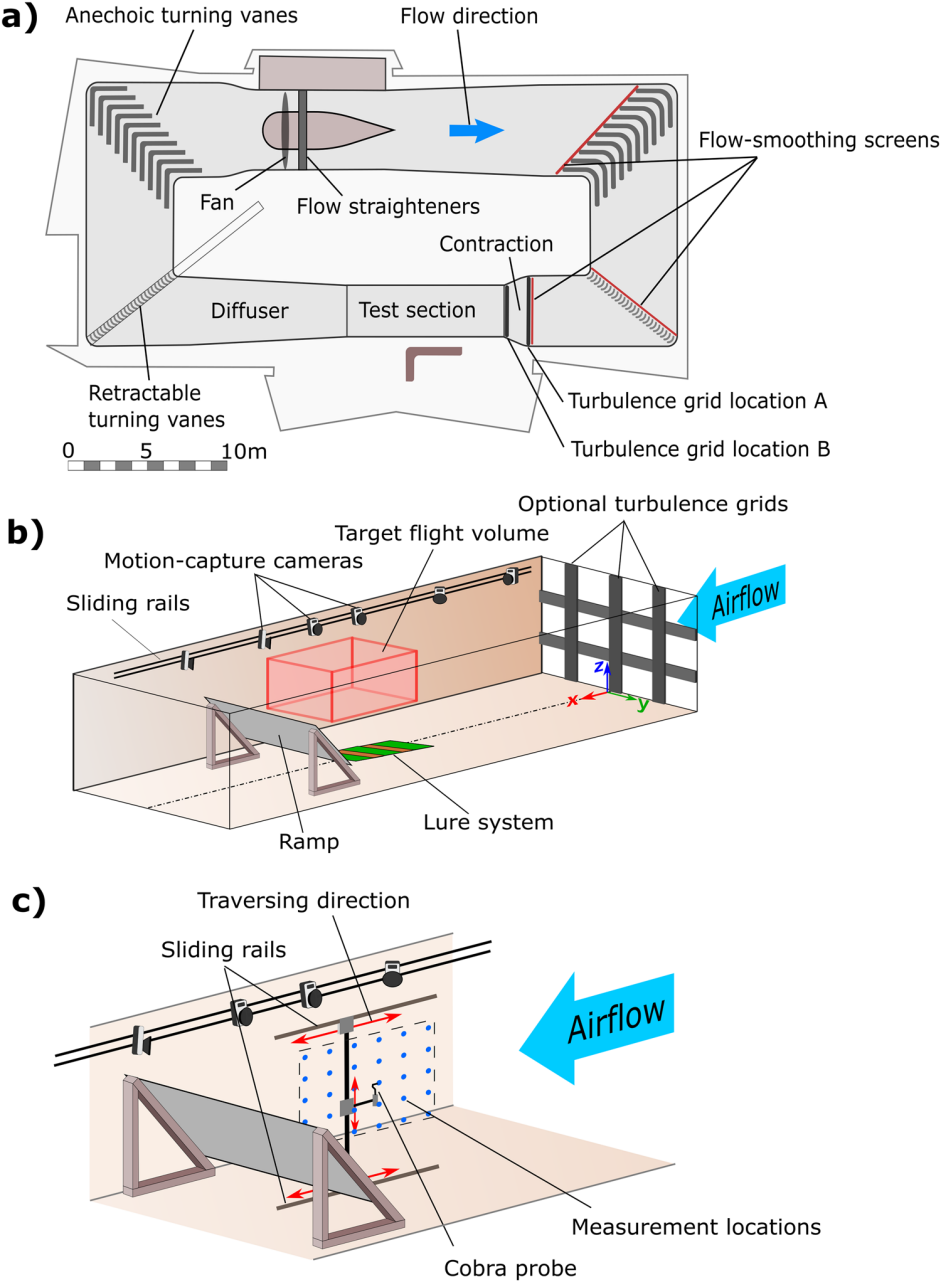
The RMIT Industrial Wind Tunnel used to facilitate soaring and windhovering flight. (**a**) A schematic of the RMIT Industrial Wind Tunnel, adapted from Ravi^19^. (**b**) The test section configured to facilitate soaring and windhovering birds. (**c**) The experimental setup used during flow mapping.

A simplified illustration of the RMIT Industrial Wind Tunnel is provided in Fig. 2a. The tunnel has a closed-circuit configuration powered by a 225KW thyristor-controlled DC motor driving a six-blade single-stage axial fan. The test section speed can be infinitely varied from 0 to ~ 50 m/s and is measured using a Pitot-static tube connected to an MKS differential pressure measuring system. The tunnel has a contraction ratio of 2:1, and very low acoustic noise is present in the test section due to anechoic turning vanes. The wind tunnel has three flow-smoothing screens at the locations indicated in Fig. 2a. In this “baseline” configuration it has nominally smooth flow in the test section (turbulence intensity of 0.8%). Modifications to the tunnel for this study included the ability to increase the turbulence levels to those found outdoors and the generation of an updraft suitable for sustained soaring flight See Fig. 2b for a diagram showing the tunnel test section configured with these changes.

### Flow mapping

A target flight volume was defined, providing a region for flow mapping efforts, as well as a region on which to focus cameras for data acquisition (as shown in Fig. 2b). The flight volume was 1.35 m long, 1.5 m wide and 0.75 m high, and had a centroid located 1.325 m above the tunnel floor and 7.125 m downstream from the inlet.

**Figure 3:**
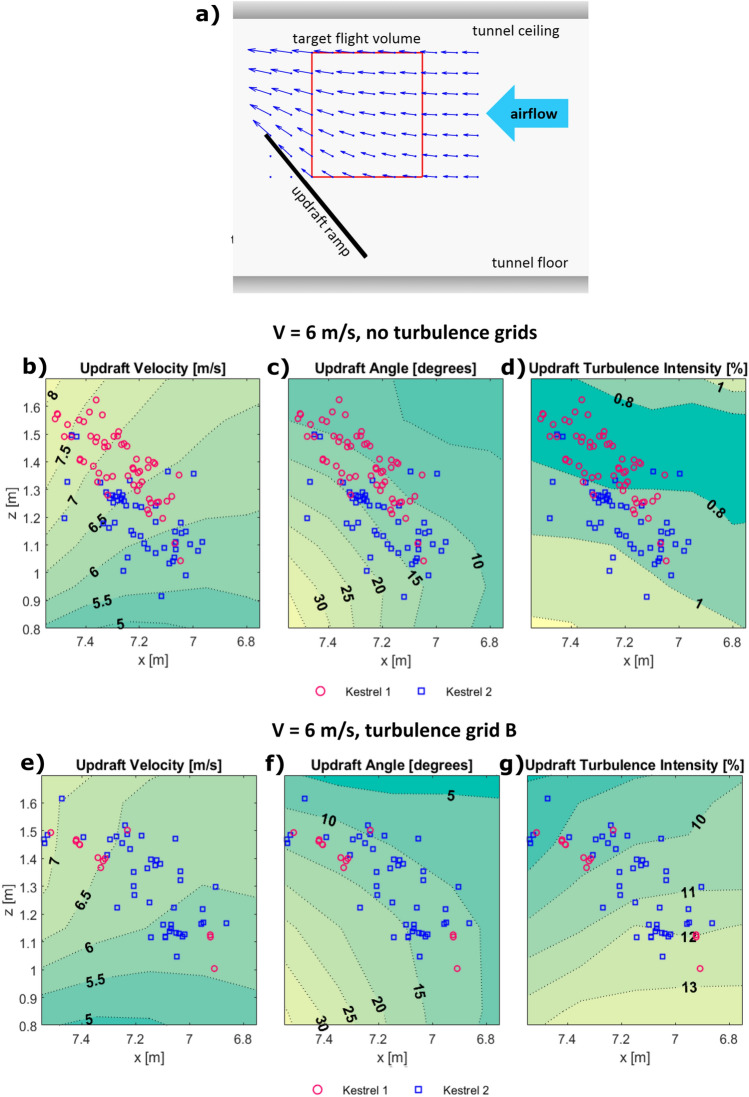
Results of flow mapping and flight testing conducted at a tunnel speed of 6 m/s. (**a**) A scale diagram depicting the relative positioning of the flight volume and ramp within the wind tunnel. Blue arrows indicate the measured flow velocity vectors at measurement locations shown in Fig. 2c. A silhouette indicates the size of a kestrel. (**b**–**d**) Contours of the flow velocity, updraft angle and turbulence intensity without turbulence grids present. Markers indicate the location of the mantle of each kestrel during a hanging flight. (**e**–**g**) Contours of the flow velocity, updraft angle and turbulence intensity with turbulence grid B installed.

Mapping of the flight volume was conducted by using arrays of point measurements from a TFI Cobra Probe mounted on a system of sliding rails as depicted in Fig. 2c. Mean flow angle, velocity and the turbulence intensity were calculated at each location from one-minute samples. The xz plane located at the centre of the test section was mapped using a square grid with 0.15 m spacing. The root of the velocity vectors in Fig. 3a represents mapping locations and shows their relative positioning within the test section. Mapping was conducted with and without turbulence grids and at tunnel speeds of 5, 6 and 7 m/s. Cobra probes were calibrated by the manufacturer TFI. Post calibration probe velocity measurements are accurate to within ± 0.3 m/s and angle measurements are accurate to within ± 1°^36^. Cobra probes measurements have previously been validated in various flow conditions^37–39^. Cobra probes have a cone of acceptance of ± 45°, and data falling outside this range is rejected. During testing, zero samples were recorded outside of this range, so all data recorded was deemed reliable.

### Updraft generation

An updraft was created by placing an obstacle to the flow on the floor of the wind tunnel test section. Several obstacle geometries were trialled; however, a ramp was found to produce the most desirable updraft characteristics. The ramp used had a surface length of 1.215 m and was inclined at an angle of 52°. The leading edge of the ramp was positioned 7.14 m downstream from the test section inlet and raised 0.145 m above the tunnel floor allowing the test section’s boundary layer to pass underneath maintaining smooth flow over the ramp.

### Turbulence generation

Turbulence was generated using grids developed during prior studies relevant for road vehicles and UAVs^40–42^. The grids consist of flat panels of different widths and spacings and by varying the size and spacing of the grid, the turbulent properties (length scale and intensity) can be controlled. The wake immediately downstream of the grids is dominated by vortices shed from the grids, however these decay into homogeneous isotropic turbulence as the flow travels downstream. Therefore a long test section is required to achieve well-mixed turbulence. The turbulence length scale and intensity likewise decay as downstream distance from the grids increases.

Two grid configurations studied by Ravi^40^ were used in this study. Both grids are comprised of 0.275 m wide flat panels arranged in a 1 m square spacing with openness ratio of 0.49. The grids have been labeled A and B based on the locations indicated in Fig. 2a. Grid A is located at the entrance of the contraction chamber and produced turbulence with a longitudinal turbulence intensity of 7.3% and longitudinal length scale of 0.22 m at a point 7.75 m downstream from the test section inlet and 1.3 m above the tunnel floor. Grid B was located at the test section inlet and produced a turbulence intensity of 12.6% and a longitudinal length scale of 0.31 m at the same location. These turbulence intensities are in the range of those measured in outdoor environments at windhovering locations. The length scales are small relative to those of the atmospheric boundary layer, however, they will nevertheless provide significant perturbations to birds or aircraft of a similar scale^43^.

### Flight trials with live kestrels

Ethics approvals for flight testing with live birds were obtained from the RMIT Animal Ethics Committee (approval number 1804) and the Victorian State Government Department of Environment, Land, Water & Planning (approval number 10008735). Flight testing was conducted in accordance with relevant guidelines and regulations. Testing was conducted in accordance with applicable ARRIVE guidelines.

### Kestrels

Two nankeen kestrels (*Falco cenchroides*) were selected for flight trials. Both birds were females, were raised in captivity, and were already trained to fly indoors. Fine mesh netting was installed upstream of the test section to ensure the rest of the tunnel was inaccessible to the birds. The birds were food motivated, and the granting or withholding of rewards was used to train them to fly in the manner and location desired. Rewards were attached to falconry lures that were concealed beneath a cover on the test section floor. After successful windhovers the baited lures were pulled into the open, allowing the birds to access their rewards. Testing to demonstrate the suitability of the wind tunnel to support hanging kestrel flight was conducted at a tunnel velocity of 6 m/s, with turbulence grids B installed.

### Motion capture

An array of 13 Qualisys Oqus 7+ infrared motion-capture cameras and three Miqus M5 optical cameras were mounted along the walls of the test section to capture the position of hemispherical reflective markers (with a diameter of 1.5 mm and 3 mm) placed on the bird’s feathers using double-sided adhesive tape. The distribution of the markers can be varied, however, for this study markers were placed on the centre of the kestrel’s head and mantle, and used to identify and record the location of hovers during flight trials. The motion capture system was calibrated daily using a measurement wand (length 297.6 mm). After calibration the standard deviation of the wand was less than 0.4 mm.

A MATLAB script was written to identify periods of windhovering flight. The script analysed head trajectory data, searching for regions in the data where head trajectories remain stationary for periods of 0.5 s or greater. After tuning, the standard deviation of head translations for each hover identified were less than 1 mm. Multiple hovers were taken from a single flight, as birds would typically windhover briefly in several locations during a single flight, before each flight was terminated with a reward. Head markers were used for windhover identification because windhovering is a flight behaviour where birds attempt to maintain stationary head positions with respect to the ground.

## Results and discussion

Characteristics of the updraft measured at the central plane (y = 0) are presented in Fig. 3. Figure 3a is a scale diagram depicting the relative positioning of the flight volume and ramp within the test section, the updraft velocity vectors in the mapped volume, and a silhouette indicating the relative size of a kestrel. The root of each velocity vector indicates a measurement location used during flow mapping. Figure 3b–d contain contour plots displaying the flow velocity, updraft angle, and turbulence intensity in the centre of the test section at a tunnel speed of 6 m/s, without turbulence grids installed. Markers indicate the position of reflective markers located on the mantle of each kestrel during hanging windhovers in the conditions described. Figure 3e,f contain contour plots of flow properties and windhover locations when the turbulence grid B was installed.

Comparisons between mapping conducted at tunnel speeds of 5, 6, and 7 m/s found little variation in updraft characteristics. When normalised by tunnel velocity, updraft velocities differed by less than 2%. Updraft angles differed by less than 0.5°.

In the baseline configuration (no grids) the tunnel flow was nominally smooth (turbulence intensity of typically ~ 0.8%). With grid A installed, the turbulence intensity ranged from 5 to 7.5% in the flight volume. Grid B produced a turbulence intensities between 8 and 13%. In both cases, the highest turbulence intensities were measured closest to the surface of the ramp due to the presence of a developing boundary layer.

**Figure 4:**
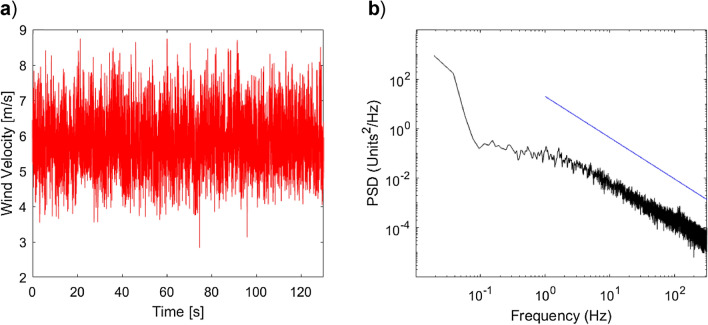
Temporal and spectral characteristics of wind measured in the RMIT Industrial Wind Tunnel configured to facilitate soaring flight. Turbulence grid B is installed, resulting in a turbulence intensity of 11.5% where birds have been observed to windhover. The freestream velocity is 6 m/s, and the measurement location is (x,y,z) = (7.15, 0, 1.3) m. (**a**) shows a 130 s sample of the fluctuating wind velocity measured by a TFI Cobra Probe. (**b**) shows the power spectral density of the flow follows the − 5/3 Kolmogorov spectral decay depicted by the blue line.

Figure 4a shows a 130 s sample of raw velocity data gathered in the wind tunnel at a freestream velocity of 6 m/s, and with turbulence grids B installed. The sample was taken at a position where birds are likely to hover. The turbulence intensity for this sample is 11.5%. Figure 4b shows the spectral density for this data. The blue line shows the − 5/3 Kolmogorov decay for mixed turbulence, which closely matches the data at frequencies above ~ 6 Hz. At 6 Hz. Thus, for eddy length scales less than 1 m, the turbulence may be considered well mixed. This encompasses the relevant eddy length scales for birds and aircraft with a chord length of ~ 0.1 m.

### Updraft characteristics

The updraft velocity and angles produced in the wind tunnel are representative of those reported for gliding birds in the literature (see Table 1). Updraft angles between 1 and 42° were measured, with the angles between 4.5 and 10° passing through the centre of the mapped volume. The tunnel was set to 6 m/s for the flight tests, which produced local updraft velocities between 5 and 8 m/s in the region of interest. This encompasses the lower gliding velocities of all but one of the birds listed in Table 1. The tunnel could be set to higher velocities to allow the analysis of gliding flight at higher airspeeds, however new flow mapping would likely be required.

### Flight results

The flight results in Fig. 3b–g confirmed that the tunnel configuration does indeed provide conditions suitable for windhovering and soaring flight within the target volume, at both low and high turbulence levels. The kestrels successfully exhibited hanging flight across a range of local speeds and updraft angles. The distribution of hover locations is qualitatively similar to the distribution of gulls flying in updrafts created by buildings^44^.

Local updraft angles for each hover location were interpolated from the mapped contours using linear interpolation from the flow mapping data grid. The mean updraft angle at hover locations was 10.4 ± 2.0 deg s.d., n = 63, for kestrel 1, and 14.1 ± 2.7 deg s.d., n = 50 for kestrel 2 in smooth flow, and 12.4 ± 1.15 deg s.d., n = 15, for kestrel 1, and 12.4 ± 2.0 deg s.d., n = 47 for kestrel 2 in turbulent flow. Most flights occurred at updraft angles greater than those reported for kestrels by Videler and Groenewold (5–8°)^4^. The wind speeds at hover locations during our testing were relatively low (6.6 ± 0.8 m/s s.d., n = 163) compared to those *estimated* by Videler and Groenewold (7–12 m/s)^4^. Turbulence characteristics cannot be compared as Videler did not record them. It is important to note that Videler’s study estimated wind conditions at kestrel’s windhovering locations based on mean wind profiles calculated from prior measurements. This provides a rough estimate of the wind at hover locations, but cannot be relied upon to provide accurate values. The controlled flow of the wind tunnel in this study provides far greater confidence of wind conditions at hover locations.

## Concluding remarks

The airflow conditions required for soaring and windhovering flight in nature were reviewed and reproduced via modifications to the RMIT Industrial Wind Tunnel. Flight trials with nankeen kestrels confirm that this facility is suitable for the study of soaring and windhovering bird flight.

Using this method existing wind tunnels may be easily configured for the study of continuous soaring bird flight, providing a simpler alternative to the construction of dedicated wind tunnels with tilting test sections. Tunnels equipped in this manner may be used to study gliding birds in smooth flow, mixed turbulence or discrete gust conditions. Interest in studying bird flight in controlled flight conditions has been rapidly increasing. This methodology provides a means to conduct studies investigating bird behaviour, the aerodynamic performance of gliding flight, or the control responses used by birds to mitigate gusts or turbulence. The facility could also be used to test the flight performance of soaring UAVs in simulated atmospheric turbulence.

## Data Availability

The datasets generated during and/or analysed during the current study are available from the corresponding author on reasonable request.
